# Structural characteristics and proximity comparison of China’s urban innovation cooperation network

**DOI:** 10.1371/journal.pone.0255443

**Published:** 2021-07-30

**Authors:** Yingying Yuan, Zenglin Han

**Affiliations:** 1 School of Geography, Liaoning Normal University, Dalian, Liaoning, China; 2 Center for Studies of Marine Economy and Sustainable Development, Liaoning Normal University, Dalian, Liaoning, China; Northeastern University (Shenyang China), CHINA

## Abstract

How to promote and improve the level of urban innovation cooperation is a major issue in China’s current high-quality economic development. Thus, enhancing innovation ability is essential to achieving high-quality economic growth under the "new normal". Based on the data of Chinese invention patents from 1985 to 2017, this paper analyzes the characteristics of China’s urban innovation cooperation network and the different roles of proximity by using social network analysis and exploratory spatial data analysis methods. The analysis results show that: (1) On the whole, the development of China’s urban innovation cooperation network is characterized by stages (initial development stage, rapid development stage, and gradual decline stage); The urban innovation cooperation network has strong connectivity and centripetal concentration but its imbalance needs to be further improved; The degree of urban participation has gradually increased, consolidating the stability of the network structure. (2) The centrality of urban innovation cooperation network has obvious characteristics of administrative center orientation, coastal areas orientation, and ‘strong east and weak west’; Beijing is the center and bridge of the network, and the network flattening characteristics are obvious; A hierarchical ‘core-edge’ structure is gradually formed for the urban innovation cooperation network, and the pyramid structure with Beijing standing at the top is being consolidated. (3) The geographical proximity presents a significant global spatial positive correlation, while the network proximity and pure network proximity have a more significant global spatial negative correlation; The local spatial autocorrelation of China’s urban innovation cooperation system based on network proximity is more obvious and identifiable than that based on the geographical proximity, which better reflects the new development model of "relationship economy".

## Introduction

The report of the 19th National Congress of the Communist Party of China points out that China’s economy has changed from high-speed growth to a new stage of high-quality development, and the driving force of economic growth needs to change from factor-driven to innovation-driven. According to the new economic growth theory, innovation is an important driving force to promote economic growth and improve living standards. Thus, enhancing innovation ability is essential to achieving high-quality economic growth under the "new normal". The rise of a new round of scientific and technological revolution and industrial change in the world as well as the in-depth implementation of China’s innovation-driven development strategy provides opportunities for China’s urban innovation and development. The city is the subject of implementing the innovation-driven development strategy and leading the regional innovative development. A city with a large number of universities, scientific research institutions, high-tech enterprises, and talents can become the main birthplace of new ideas and technologies [1] and the most active and abundant unit of innovation activities.

With the development of globalization, informatization, and networking, the relationship between cities becomes closer, and the central place theory emphasizing unilateral vertical connection provokes debate [2]. Some scholars believe that urban innovation is no longer "local innovation", and a city is an important node in the urban network system. Also, the spatial structure of the urban network system has gradually changed from a hierarchical pattern to a networked one [3–5]. The research of the network paradigm has become a new field of urban research [6,7]. As for the urban research on network paradigm, knowledge and technology exchange is no longer limited to a fixed space, and more emphasis is put on interactive learning in urban space of flows through cooperation or other joint activities [8]. The coexistence of " space of places " and "space of flows" promotes the reconstruction of innovation network space with the city as the hub [9]. Based on this, the traditional urban hierarchy and individual scale attribute research cannot truly reflect the evolution characteristics of China’s urban network [10].

With the quantification of inter-city interaction, the research on urban relations is increasingly diversified. The Globalization and World Cities Study Group and Network (GAWC) is a leader of network paradigm research. They used the institutional address of advanced producer service enterprises to construct the world city network and investigated the modern service relationship between the cities [7]. Inspired by GAWC, the flow data such as population movement [11–13], traffic connection [14–16] and enterprise cross-city connection [17,18] have been widely used in the study of urban networks. As the world enters the era of knowledge economy, innovation and development have become the theme of The Times. The city-centered innovation network space with cities as the hub is gradually rising, and the knowledge exchange and innovation cooperation between cities are becoming increasingly frequent. Therefore, exploring innovation cooperation between cities has become a new requirement of urban network research.

The discussion of urban innovation cooperation is still in its initial stage, and the difficulty lies in data selection and how to build urban innovation connections. Existing researches mainly focus on the following three aspects. First, the indicators representing the innovation capacity of cities and the gravity model are used to construct the innovation network, but this indirect simulation of attribute data can hardly truly reflect the actual innovation connection between cities. Secondly, the data of talent flow is used to build the urban innovation network. However, due to the imperfection of the talent information database China, the timeliness and continuity of data collection are insufficient. Finally, the distribution data of innovative enterprise institutions [19], patent transfer [20], patent cooperation [21] and scientific research paper cooperation [22] are used to construct the urban innovation network. However, the existing research usually takes a specific year or specific industry, such as the equipment manufacturing industry [23] and biotechnology industry [24] as the research object. Also, the research on the network structure characteristics and spatial and temporal evolution of the long-term series of the urban innovation network in China is slightly insufficient. For example, Huang et al. used the headquarters-branches of enterprises to explore the connection characteristics of China’s urban network, but a comparative analysis of the evolution characteristics of the network is lacked [19]. Besides, the network connection characteristics of general cities cannot be measured by a specific industry, which is difficult to fully reflect the network pattern of Chinese cities. In addition, the innovation ability of cities includes the improvement of both internal and external network connections, and they interact with each other to provide the supplement of technology and knowledge [25]. Therefore, focusing on the agglomeration effect of the urban internal network and the complexity of the external network helps to clarify the internal and external focus of urban competitiveness. Most of the existing studies focus on the external relations of cities. In the analysis of network structure, centrality, such as degree centrality, strength centrality, closeness centrality, and betweenness centrality, is exploited to explore the external cooperation ability of cities, but the importance of intra-city cooperation is neglected. Therefore, this paper introduces "self-contained centrality" to represent the internal competitiveness of a city.

At the end of the 19th century, the proximity theory proposed by the French Proximity Dynamics School became the research basis of knowledge exchange and spillover mechanism [26]. Krugman believes that intangible knowledge flow will not leave a written trace that can be measured and traced [27]. Jaffe thinks that knowledge flow leaves some written records (e.g., patents) because patents contain detailed geographic information of inventors and can be traced [28]. The invention subjects of patents tend to cluster in space and can be divided into two forms: geographical proximity cluster and network proximity agglomeration. In a geographical proximity cluster, the innovation subjects can communicate face to face, and knowledge spillover and labor flow can promote the innovation development of the cluster [29,30]. Clusters can affect the surrounding areas through the spatial diffusion effect. Geographical proximity is crucial in the diffusion process [31], and knowledge spillover takes the form of hierarchical diffusion. It has been indicated by a large number of empirical studies that knowledge spillover is limited by geographical scope. Specifically, the advantage of geographical proximity conforms to the law of geographical distance attenuation, and a shorter distance is conducive to the exchange of invisible knowledge and information [32,33]. Since the positive externality of knowledge is usually transmitted through face-to-face contact [34,35], the farther the distance, the smaller the positive externality of knowledge. Marshall once proposed that "secrets of industry are openly distributed in the air" to emphasize the importance of knowledge spillover and geographical proximity when enterprises gather [36]. In the study of innovation cooperation, Liu also verified the importance of geographical proximity in interurban technology transfer in Northeast China. However, some scholars believe that excessive geographical proximity can easily cause technology lock-in, spatial lock-in, and lack of flexibility, and there is a "self-fertility" cluster risk [37]. The network proximity agglomeration means that geographically distributed clusters can communicate, exchange, and cooperate through the network. In the diffusion process, network proximity is crucial, and knowledge spillover has a leaping form of a complex network [38]. As for the network proximity, the actors may not be adjacent geographically but they are directly related in the cooperative network. With the development of economic globalization, innovation subjects can exchange knowledge and information across regional boundaries, thus forming a cross-regional cooperation network [39–41]. The relevant research shows that network proximity helps the innovation subject to break the regional lock-in and obtain new information and knowledge outside the region to achieve sustainable innovation [42,43]. Lee [44] pointed out that network proximity better defines the biotechnology co-patenting relationships among the U.S. cities compared with geographical proximity.

Synthesizing the above analysis, this paper is different from the existing research in the following aspects: (1) A new perspective of urban innovation cooperation patterns based on patent cooperation data is proposed. The patent data used in this paper are the licensed patents with novelty, innovation, and practicability that have been examined by the China National Intellectual Property Administration. Different from the previous studies of innovation cooperation based on patent data, the licensed patent data has a higher quality, which is conducive to our research on the innovation networks of Chinese cities in the context of high-quality development. Besides, different from the related research on innovation cooperative networks, this paper not only reveals the characteristics of innovation networks but also analyzes the evolution of innovation networks in different years and stages. (2) A new perspective for the comparative study of proximity is presented. Different from the previous proximity studies based on the negative binomial regression model [45], this paper exploits the Exploratory Spatial Data Analysis(ESDA) method to explore the role of two agglomeration forms in China’s urban innovation cooperation, which provides a useful supplement for the research on multi-dimensional proximity.

The rest of this paper is organized as follows: the data and research methods used in this paper are introduced in the following section. Section 3 summarizes the characteristics of the network structure in China’s urban innovation cooperation from the perspectives of overall characteristics and individual characteristics. Section 4 analyzes the differences between network dependence and geographical proximity dependence of urban innovation cooperation networks. Finally, the research conclusions of the urban innovation cooperation network and the implications of China’s innovation cooperative network are given.

## Materials and methods

### Data

**Fig 1:**
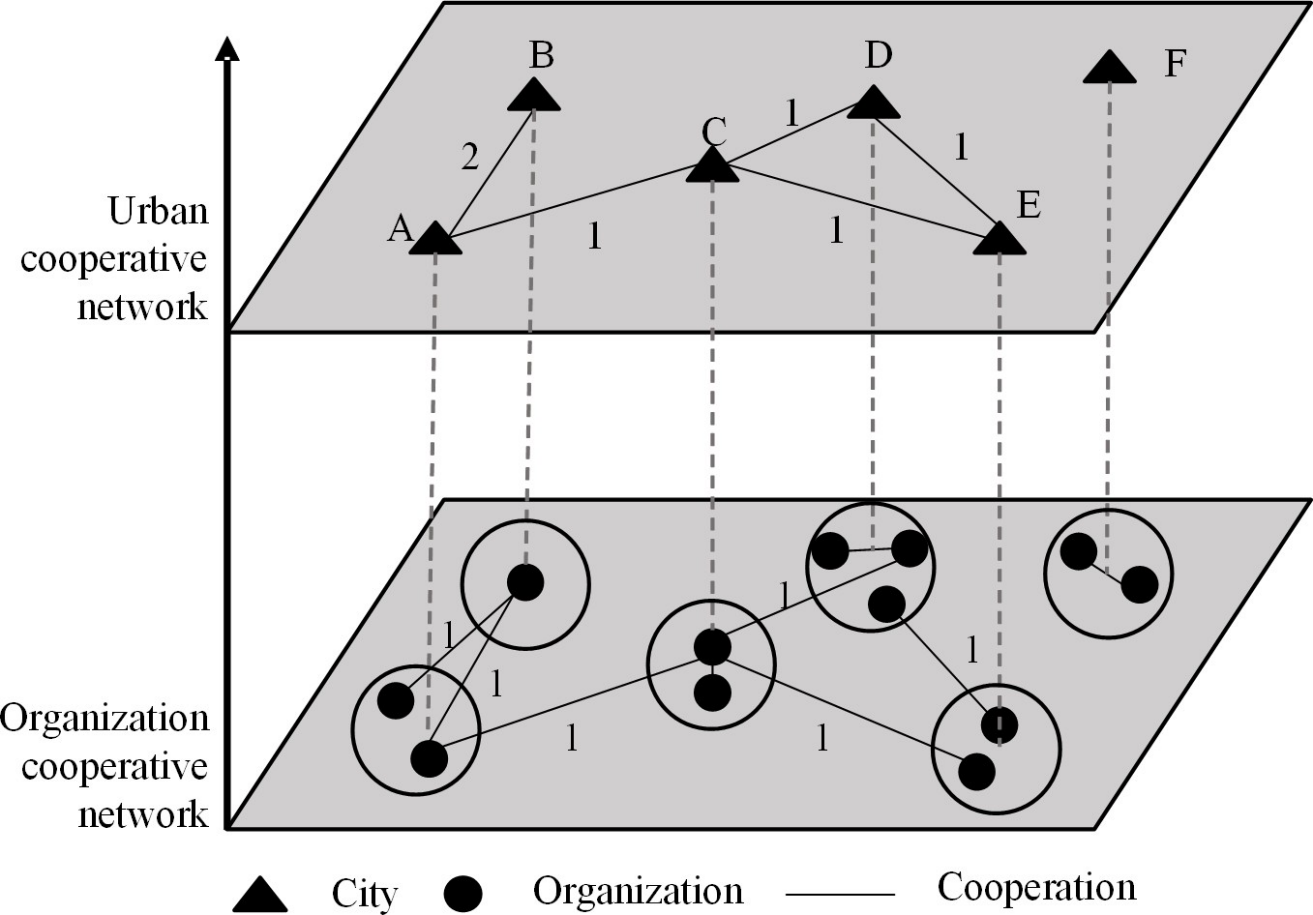
A model of patent-city cooperation network translation.

The patent data of this study was obtained from the incoPat Technology Innovation Information Platform (https://www.incopat.com). China uses a patent application authorization system, and the patent application does not mean a patent grant. To meet the authorization requirements and obtain an authorization certificate with legal effect, a technology patent must pass an examination by the State Intellectual Property Office. To investigate the high-quality patent cooperation network, this study only used the data of authorized patents for co-inventions. Considering that it generally takes 18 to 36 months for a patent application to be authorized, the scope of the data collection was set to 1985 to 2017 to ensure completeness of the dataset. In the data process to screen cooperative patents, the individual patent applications without identifiable addresses were eliminated. To construct an undirected weighted network, the cooperative organizations are first merged according to the cities at the prefecture level and above, and a total of 336 cities are included. Then, and the cooperative organization network is translated into the urban cooperative network, which is shown in (Fig 1).

### Methodology

#### Social network analysis

This paper mainly follows the social network analysis method to analyze China’s urban innovation cooperation network from the overall network characteristics and individual network characteristics. Specifically, the overall network is characterized by network density, network centralization, and network heterogeneity. Meanwhile, network degree centrality, strength centrality, self-contained centrality, closeness centrality, and betweenness centrality are taken as the individual network characteristic indices to study the status and role of cities in the network [46]. The specific calculation formula and meaning of these indices are listed in Table 1.

**Table 1:** Explanation of main indicators for characteristics of city co-invention network.

Index	Formula		Indicator meaning
Network density	$D=\frac{l}{n(n-1)}$	(1)	The higher the density, the closer the cooperation between cities.
Network centralization	$C=\frac{\sum_{i=1}^{n}\left(C_{D}{}_{\max}-C_{D}(i)\right)}{\max[\sum_{i=1}^{n}\left(C_{D}{}_{\max}-C_{D}(i))\right]}$	(2)	The larger the network centralization, the stronger the centripetal concentration of the network.
Heterogeneity	$H={\sum_{i=1}^{n}\left(C_{D}(i)/\sum_{i=1}^{n}C_{D}(i)\right)}^{2}$	(3)	The more heterogeneous the network, the more uneven the degree distribution of the nodes.
Degree centrality	$C_{D}(i)=\sum_{j=1}^{n}a_{ij}(i\neq j)$	(4)	The higher the degree centrality of a city, the stronger the control ability of the city over other cities.
Strength centrality	$C_{S}(i)=\sum_{j\in n}W_{ij}(i\neq j)$	(5)	The higher the strength centrality of the city, the more times the city cooperates with other cities.
Self-contained centrality	$C_{R}(i)=\sum_{i=1}^{n}a_{ii}$	(6)	The higher the city’s self-contained centrality, the more cooperation within the city.
Closeness centrality	$C_{B}(i)=\sum_{j}^{n}\sum_{k}^{n}\frac{g_{jk}(i)}{g_{jk}}$	(7)	The higher the closeness centrality, the stronger the intermediary ability of the city in the network.
Betweenness centrality	$C_{C}^{-1}(i)=\sum_{j=1}^{n}d_{ij}$	(8)	The higher the betweenness centrality, the shorter the distance between the city and other cities.

Note: The indicators are obtained by reference to Freeman(1979) and collated by the authors. *n* is the total number of cities in the network; *l* is the number of cooperation actually owned; *C*_*D*_(*i*) is the centrality of city *i*; *C*_*Dmax*_ is the maximum value of network centrality; *a*_*ij*_ is the adjacency matrix of inter-city cooperation, with 1 for cooperation and 0 for non-cooperation; *W*_*ij*_ is the number of cooperation between city *i* and city *j*; *a*_*ii*_ is the number of cooperation within city *i*; *g*_*jk*_ is the shortcut number between city *k* and city *j*, *j*≠*k*≠*i*, *j*<*k*; *g*_*jk*_(*i*) is the number of shortcuts through city *i* between city *k* and city *j*; *d*_*ij*_ is the shortcut distance between city *i* and city *j*.

#### Exploratory spatial statistical analysis

The geographic autocorrelation and network autocorrelation of urban cooperation times are important indicators for understanding and comparing their spatial patterns. Geographic autocorrelation and network autocorrelation refer to the geographical correlation and network correlation of city attribute values, respectively. In this paper, Moran’s index is adopted to analyze the spatial and network autocorrelation characteristics of urban innovation cooperation. The global Moran’s I statistic is defined as:

$$I=\frac{n\sum_{i=1}^{n}\sum_{j=1}^{n}W_{ij}(x_{i}-\bar{x})(x_{j}-\bar{x})}{\sum_{i=1}^{n}\sum_{j=1}^{n}W_{ij}\sum_{i=1}^{n}{\left(x_{i}-\bar{x}\right)}^{2}}$$ (9)

where *n* is the number of cities; *x*_*i*_ and *x*_*j*_ are the strength degree of the city *i* and *j*, respectively; $\bar{x}$ is the mean of the strength degree; *W*_*ij*_ is the geographical weight ($W_{ij}^{g}$) or network weight ($W_{ij}^{n}$). Because of the dispersion of cities, this paper exploits the k nearest neighbor (KNN) algorithm to determine whether the cities are adjacent, and the default value of K is 4.

The local spatial autocorrelation method is adopted to represent the specific spatial location of agglomeration or outliers and analyze the spatial correlation and degree of difference between the city and their neighboring cities. The formula of local Moran’s I (LWI) is as follows:

$$I_{i}=\frac{n(x_{i}-\bar{x})\sum_{j=1}^{n}w_{ij}(x_{j}-\bar{x})}{\sum_{i}{\left(x_{i}-\bar{x}\right)}^{2}}$$ (10)

### Characteristics of urban innovation cooperation network

**Fig 2:**
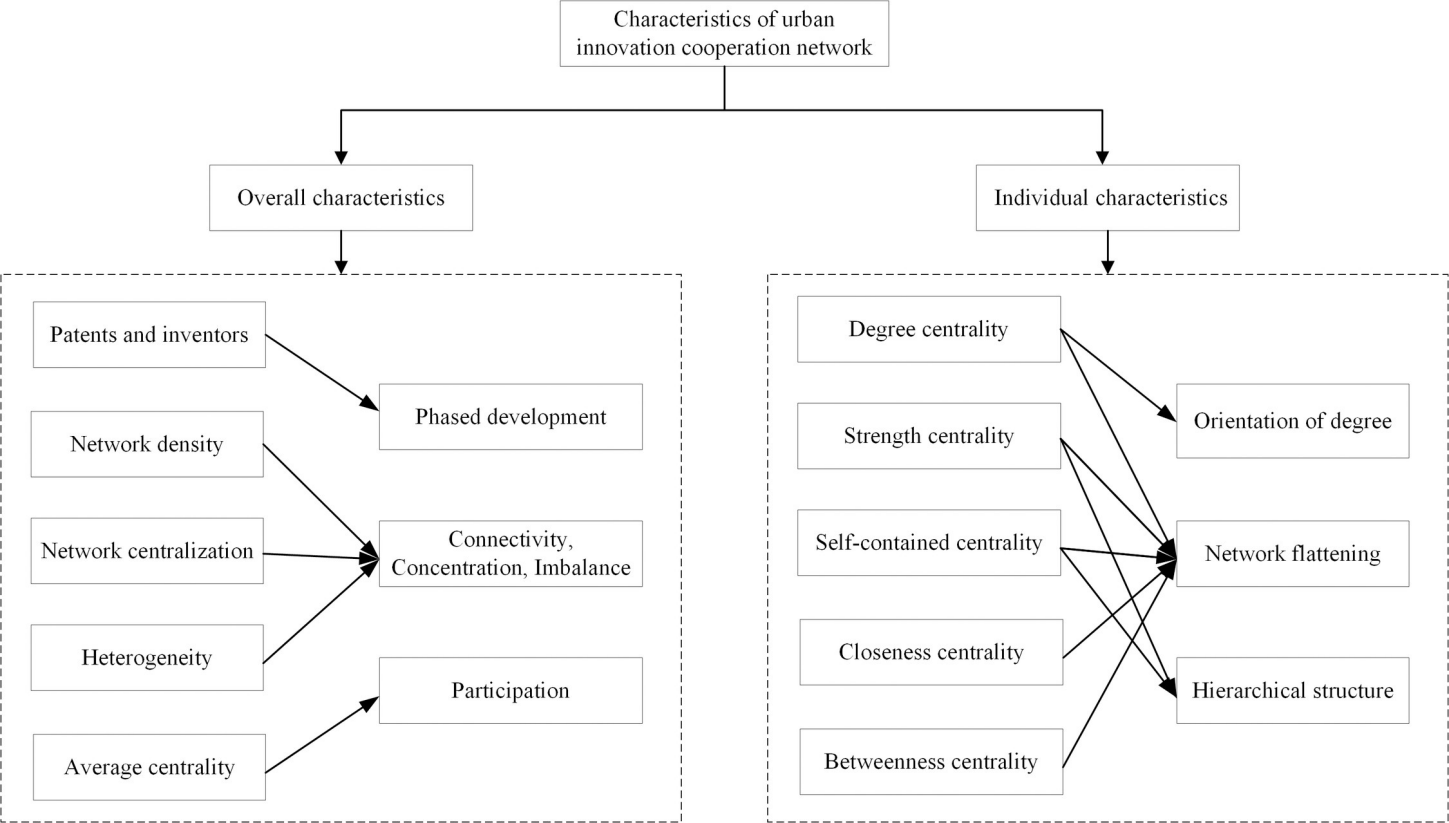
The research framework of network structure characteristics.

In this section, we first summarize the overall characteristics of the network structure of urban innovation cooperation in China. Then, the individual characteristics of the network are summarized based on the five centrality indexes. The research framework of this section is illustrated in (Fig 2).

### Overall characteristics of urban network

**Fig 3:**
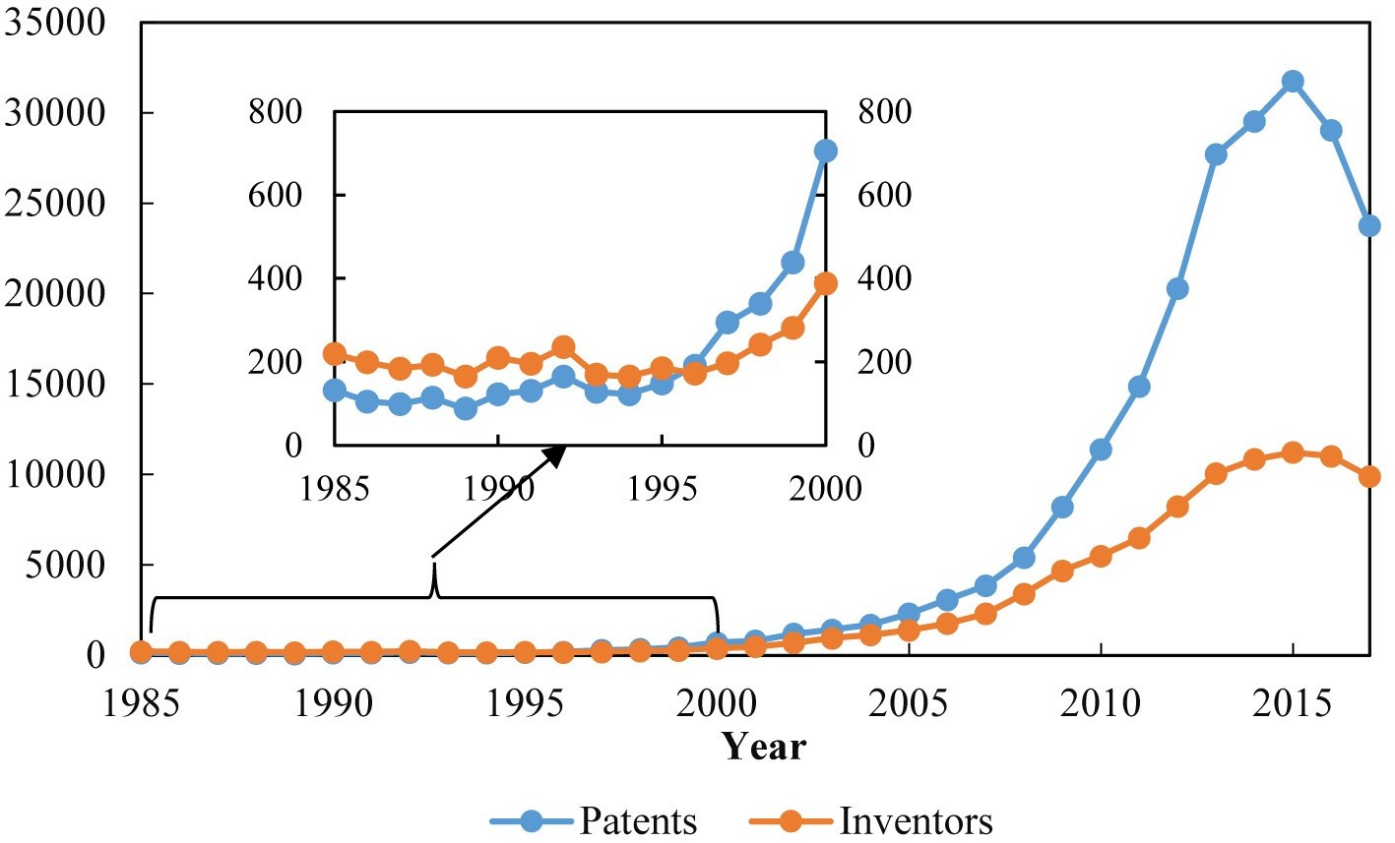
The general information of the co-invention patents and inventors in 1985–2017.

The network of urban innovation cooperation presents the characteristics of phased development. It can be seen from the statistics in (Fig 3) that the development of urban innovation cooperation in China can be divided into three stages: the initial development stage (1985–2006), the rapid development stage (2007–2015), and the gradual decline stage (2016–2017). The first patent law was promulgated in China in 1984, and patent applications began to process in 1985. Because patent authorization is in the initial stage of development, the scope of patent protection objects is relatively narrow. Meanwhile, the subject of a patent application is greatly affected by domestic political events, and the number of authorized patents fluctuates. The average annual number of authorized patents is only 626, and the average annual growth rate is only 17.04%. In 1996, the number of patents began to exceed the number of innovation subjects, indicating that although the development of patent authorization in China is slow, it shows a good development trend. As for the second stage, the National Conference on science and technology was held in 2006. The State Council issued the outline of the national medium and long-term science and technology development plan, which highlighted the importance of strengthening independent innovation and building innovative countries. In 2015, the State Council issued several opinions on deepening the reform of system and mechanism and accelerating the implementation of innovation-driven development strategy, which aimed to create a policy environment and institutional environment for mass entrepreneurship and innovation initiative. The number of authorized patents in China is developing rapidly, which increased from 3849 in 2007 to 31750 in 2015, with an annual growth rate of 30.18%. As for the third phase, the previous studies of the development stages of innovation networks reveal a rising trend. Different from these studies, this paper presents an obvious downward trend. Due to the development and implementation of the patent quality improvement project launched by the State Intellectual Property Office of China, the overall supervision of patent applications has made efforts to optimize the application structure and improve the application quality since 2016. From 2016 to 2017, the number of patent authorizations and the number of innovation subjects showed a synchronous decline.

**Fig 4:**
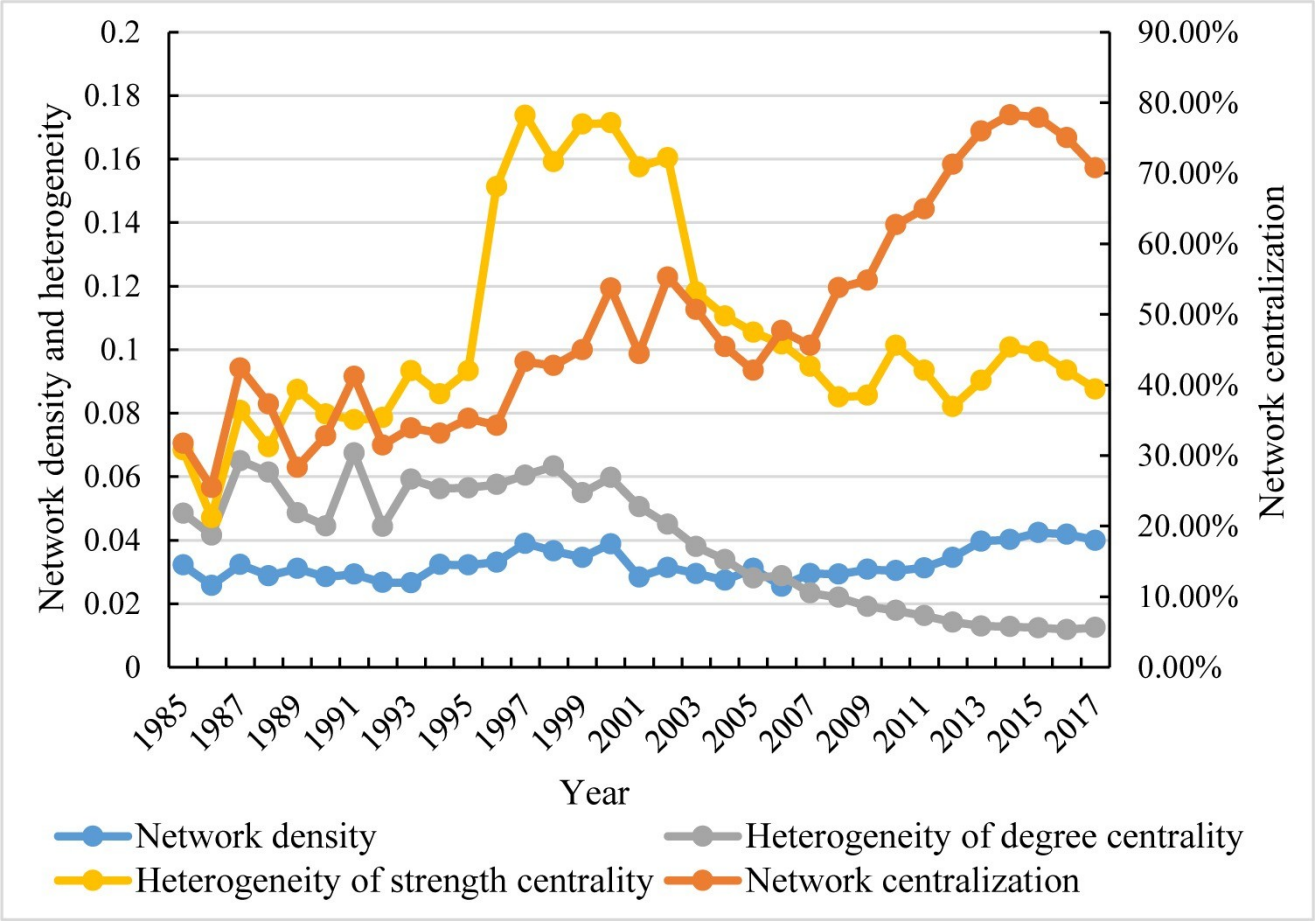
Statistics on the overall characteristics of city innovation cooperation network in 1985–2017.

The urban innovation cooperation networks have strong connectivity and centripetal concentration, while its imbalance needs to be improved (Fig 4). The average density of the binary network of urban innovation cooperation (the network formed by binarization according to whether the number of network cooperation is 0) is only 0.032, indicating low direct connectivity between cities. The network density also fluctuated from 1985 to 2006, showing a turbulent development trend; Since 2006, the network density has significantly increased and maintained at a high level, while the network connectivity has improved relatively. The network centralization of the binary network rises in fluctuation and is stable at about 70%, indicating typical centripetal characteristics of the network. After the fluctuating development, the heterogeneity of the network degree centrality gradually decreased after 2000, indicating a gradually uniform distribution of innovation cooperation between the cities. Meanwhile, the overall heterogeneity of the strength centrality presents an inverted "U" shape. From 1996 to 2002, Beijing was the dominant city in the urban innovation cooperation network, and other cities were underdeveloped. In this case, the heterogeneity of strength centrality increases obviously, and the development of network strength centrality was extremely unbalanced; After 2003, with the rapid development of Shanghai, Guangzhou, Shenzhen, and other cities, the heterogeneity of strength centrality decreased significantly. From 1985 to 2017, the heterogeneity of strength centrality was always greater than the degree heterogeneity, which indicates a balanced distribution of cooperative cities in the network. However, the number of cooperation is not balanced, showing a "quantity" improvement instead of "quality" development. Therefore, to ensure the sustainability of the improvement of the innovation capacity of Chinese cities in the future, cities should not only pursue the increase of the number of patents but also pay attention to the improvement of patent quality.

**Fig 5:**
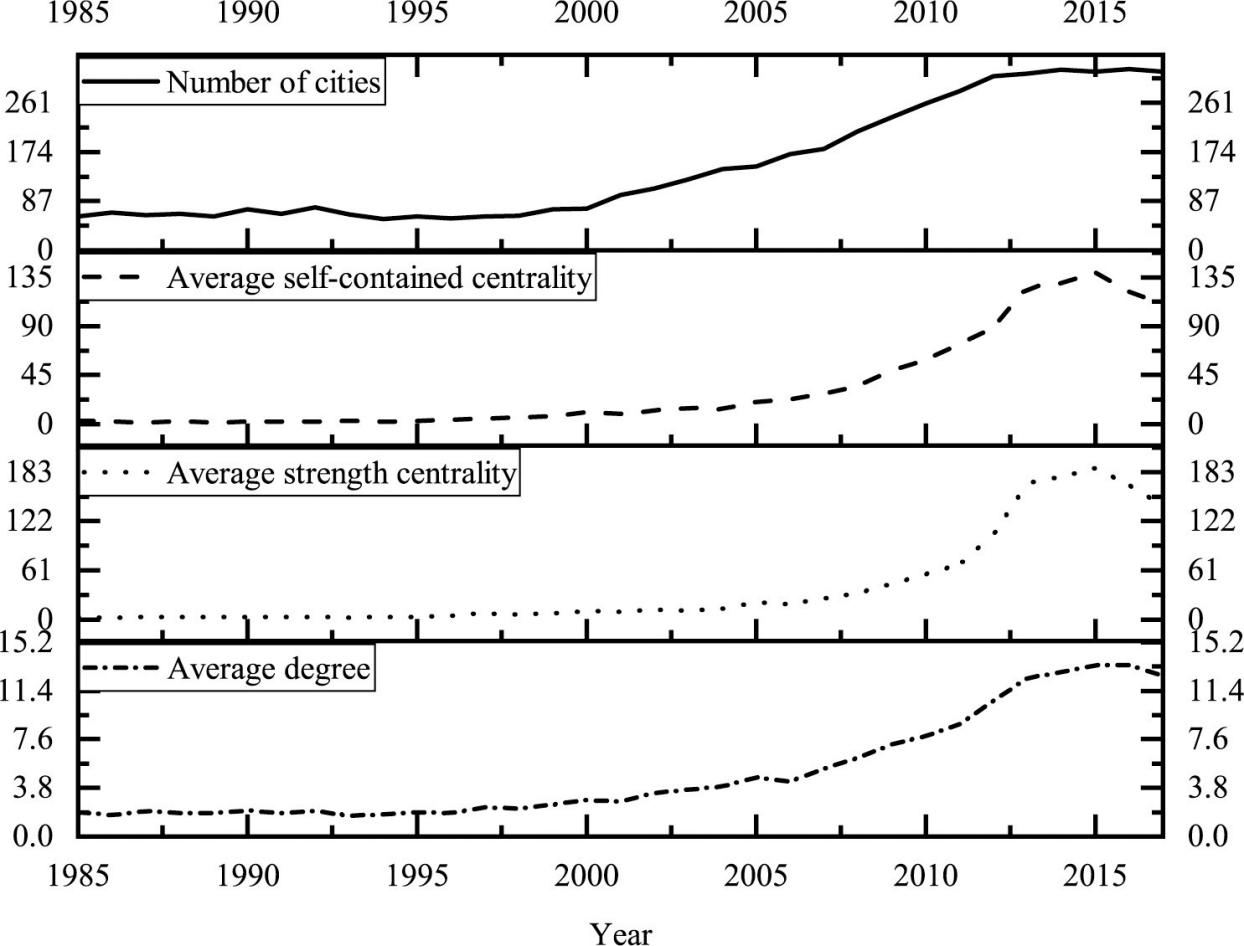
Scale and centrality of China’s urban innovation cooperation network in 1985–2017.

It can be seen from (Fig 5) that the degree of urban participation gradually increases, and the internal cooperation and external cooperation improves simultaneously. After nearly 15 years of slow development, the number of cities in the network gradually increased after 2000. Especially, after 2012, under the stimulation of encouraging innovation in China, the enthusiasm of cities to participate in innovation cooperation has increased significantly, and 91% of China’s cities at the prefecture level and above have participated in innovation cooperation. The increase of average degree further indicates the improvement of the participation degree of each city. The correlation coefficient between the average strength centrality and the average self-contained centrality is as high as 0.99. This significant positive correlation indicates that the internal cooperation and external cooperation of the city are not completely independent. Specifically, the internal cooperation of the city will improve the external cooperation of the city, and the improvement of the external cooperation will also increase the economic effect of the internal agglomeration of the city. This is consistent with the conclusion obtained by Rozenblat’s analysis of the global urban network based on the network data of enterprise subsidiaries [47]. It can be seen from the changing trend of the overall centrality that, affected by China’s patent quality improvement project in 2016, the average self-contained centrality and average strength centrality decrease, but the decline of the average degree is not obvious. This indicates that the relationship number of the urban network is gradually stable, and the network structure has been consolidated.

### Individual characteristics of urban network

**Fig 6:**
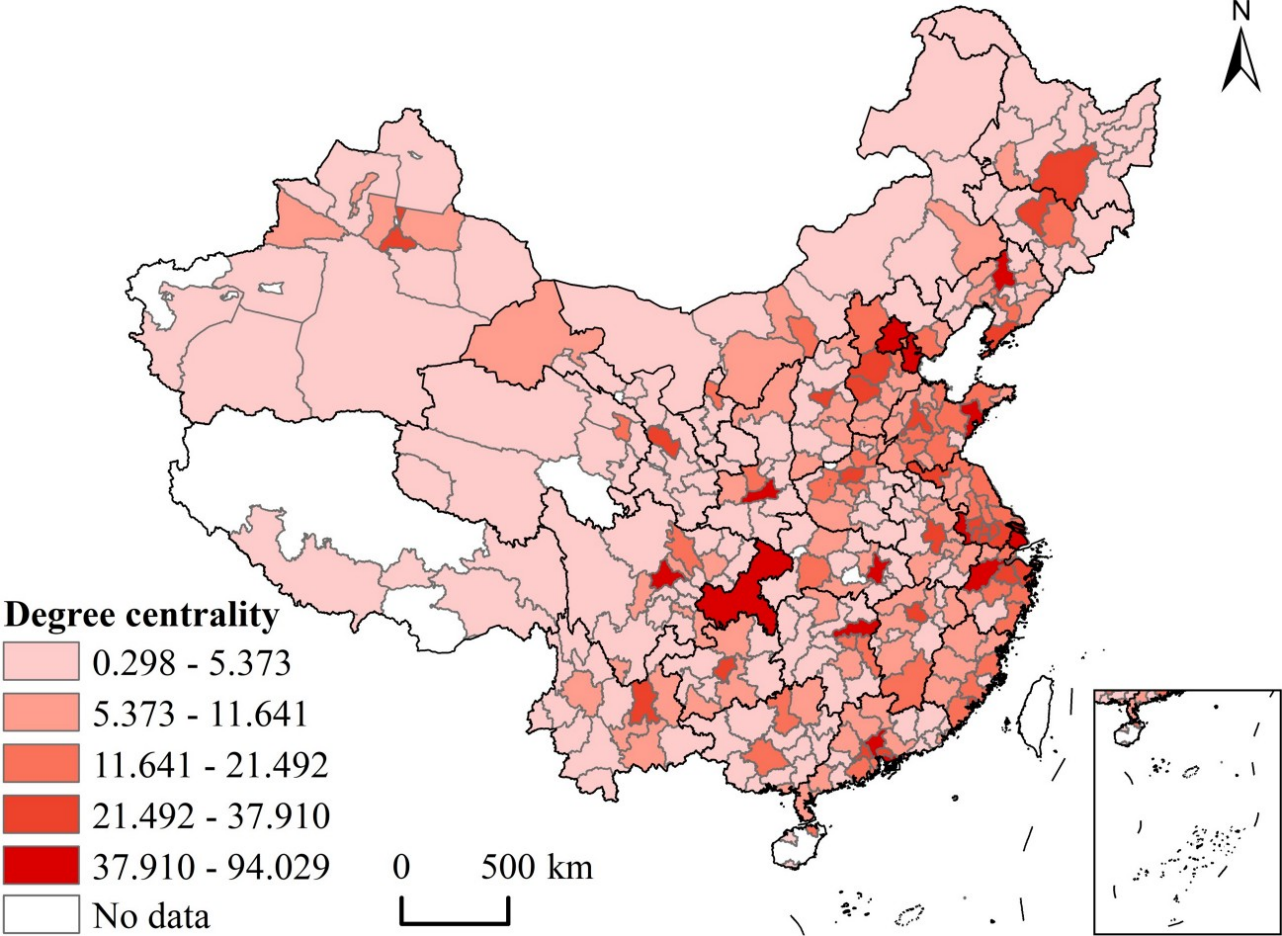
Degree centrality profiles in national city co-innovation network in 1985–2017. This map is drawn by the authors. This map was created using ArcGIS ® software by Esri, ©National Platform for Common Geospatial Information Services,Creative Commons Attribution 4.0(CC BY 4.0).

It can be seen from (Fig 6) that the centrality of the urban innovation cooperation network is strong in the east and weak in the west, showing the characteristics of administrative center orientation and coastal region orientation of the network. This result is consistent with most conventional urban network analysis. (Fig 6) shows that the centrality of cities corresponds to the administrative level of cities and the macro pattern of economic and social development. Also, the cities with higher centrality are concentrated in provincial capitals and coastal cities. Specifically, Beijing-Tianjin-Hebei urban agglomerations, Yangtze River Delta, Pearl River Delta, Hachang urban agglomeration, and Chengdu-Chongqing urban agglomeration have higher centrality, forming a more obvious spatial high-value agglomeration. The spatial distribution of city centrality shows a zonal differentiation of "strong in the east and weak in the west". The eastern coastal areas have a continuous distribution of city centrality, forming obvious spatial differentiation with western cities. The city centrality of provincial capitals such as Wuhan, Changsha, Xi’an, Zhengzhou, and Hefei in the central region is significantly higher than that of other surrounding cities, indicating the strong leading role of the provincial capitals.

As listed in Table 2, Beijing is the center and bridge of the network, and the network is flat. Also, all the centrality indicators of Beijing rank first and are far higher than those of the other cities, showing the leading position of this city in innovation capability. As a national center of politics, economy, and education, Beijing gathers abundant human resources, R & D capital, and many innovative subjects (enterprises, universities, and scientific research institutes), which provide inexhaustible power for innovation and development. Different from previous studies, this paper not only focuses on the enhancement of the external network connection of urban innovation ability but also studies the enhancement of the internal connection of cities [17]. It can be found that the cities have basically the same order of strength centrality and self-contained centrality. However, the self-contained centrality ranking of Shenzhen, Foshan, Qingdao, and other cities is higher than the strength centrality of these cities. This result indicates that the internal connection of these cities is stronger than that of the external connection, and the external network connection needs to be improved. The betweenness centrality and closeness centrality of Beijing, Shanghai, Wuhan, Nanjing, and Guangzhou are all at the forefront, indicating that the urban innovation cooperation network is flat. As the core of this network, a few cities occupy the core hub of the network in the country, which assume the functions of distribution and intermediary and master the national innovation resources.

**Table 2:** Centrality analysis of city innovation cooperation network in China in 1985–2017.

City	Degree centrality	City	Strength centrality	City	Self-contained centrality	City	Closeness centrality	City	Betweenness centrality
Beijing	94.03	Beijing	2.889	Beijing	345.528	Beijing	25.574	Beijing	94.366
Shanghai	69.851	Shanghai	0.675	Shenzhen	71.278	Shanghai	7.391	Shanghai	76.835
Wuhan	60.597	Nanjing	0.498	Shanghai	69.534	Wuhan	5.146	Wuhan	71.734
Nanjing	57.612	Shenzhen	0.471	Nanjing	44.89	Nanjing	3.901	Nanjing	70.231
Guangzhou	53.134	Hangzhou	0.312	Guangzhou	31.451	Guangzhou	3.736	Guangzhou	68.089
Tianjin	52.836	Guangzhou	0.256	Foshan	31.015	Chengdu	3.545	Tianjin	67.951
Changsha	51.94	Tianjin	0.236	Hangzhou	25.588	Changsha	3.333	Changsha	67.404
Shenzhen	51.343	Wuhan	0.203	Qingdao	20.143	Shenzhen	3.029	Shenzhen	67.269
Chengdu	51.343	Chengdu	0.187	Wuhan	15.29	Xi’an	2.906	Chengdu	67.269
Xi’an	51.343	Jinan	0.167	Tianjin	12.878	Tianjin	2.781	Xi’an	67.269

**Fig 7:**
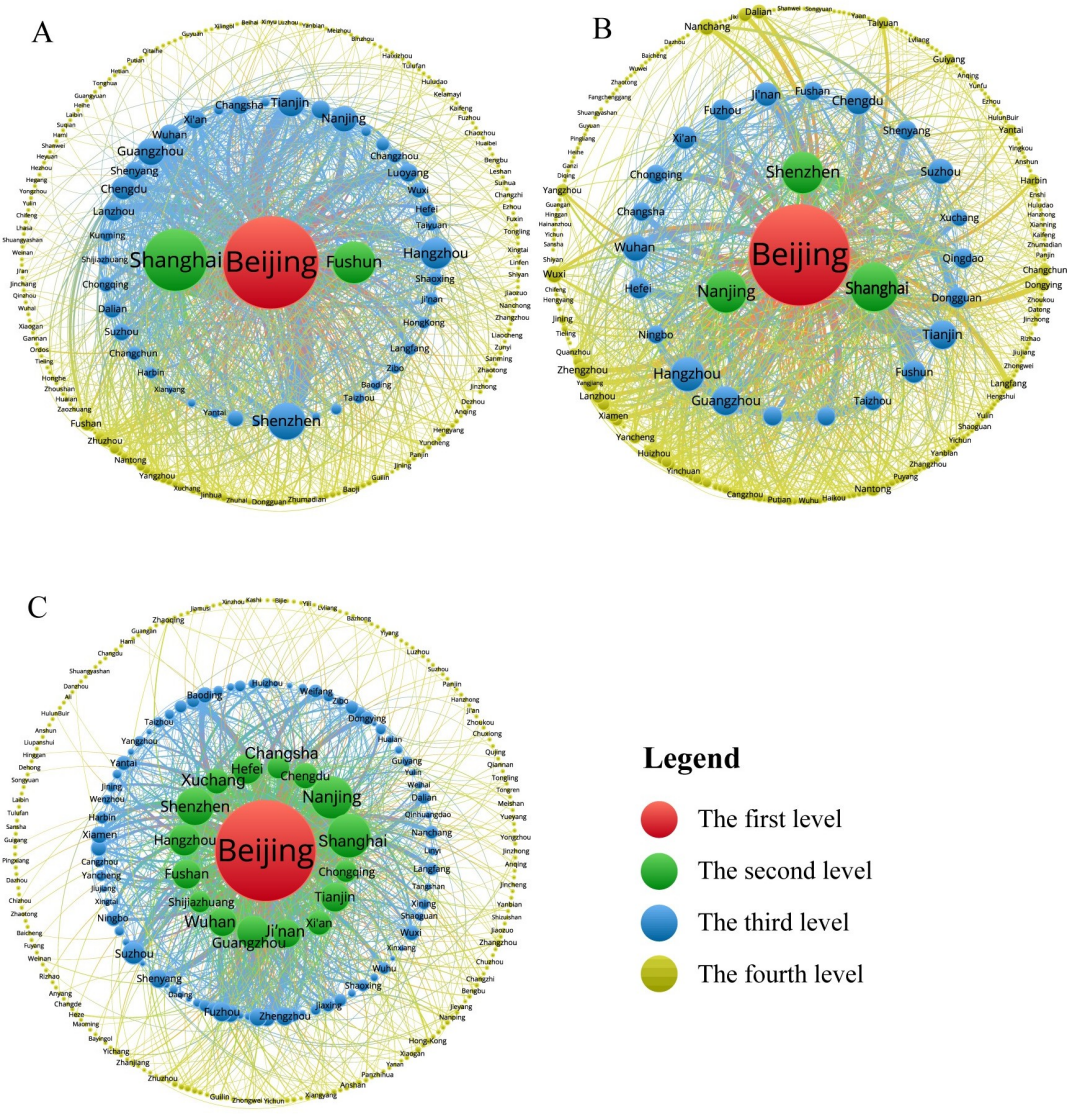
Evolution of the hierarchical structure of urban innovation cooperation network from 1985 to 2017. A. 1985–2006. B. 2007–2015. C. 2016–2017. Note: The size of the node in the figure is proportional to the strength centrality and self-contained centrality of the node. The edge thickness is positively correlated with the amount of cooperation between nodes.

The urban innovation cooperation network has a hierarchical structure, and Beijing is always at the top of the pyramid. The Pajek and Vosviewer software are employed to analyze the hierarchy of China’s urban innovation cooperation network in terms of city strength centrality and self-contained centrality. It can be seen from (Fig 7) that the urban network gradually formed a "core-edge" structure with distinct levels during 1985–2017. Specifically, from 1985 to 2006, the urban innovation cooperation network was constructed with Beijing as the primary core and Shanghai and Fushun as the secondary core. Meanwhile, the cooperation was oriented to resource-based cities. Besides, 85.3% of the cities are located on the edge of the network, and the network structure is in the initial stage of development. In addition, Fushun stands out as an important petroleum city. SINOPEC Fushun Research Institute of Petroleum and Petrochemicals and Liaoning Petrochemical University in Fushun city have close cooperation with China Petrochemical Corporation in Beijing; From 2007 to 2015, Nanjing and Shenzhen leaped from the third level to the second level of the network, and a network structure of "one dominant pole with three pillars" was formed. In the third level, Hangzhou, Guangzhou, Tianjin, Wuhan, Chengdu, and other cities followed closely, gradually widening the gap with the cities located on the edge of the network; From 2016 to 2017, the urban innovation cooperation network has developed into a typical "core edge" hierarchical structure. Beijing is still at the top of the pyramid with 50772 times of innovation cooperation, which accounts for 29.6% of the total number of network cooperation. There are 16 cities in the second level, accounting for 4.82% of the total number of cities in this network. There are 82 cities in the third level, accounting for 24.7% of the total number of cities in this network. Different from the traditional research, in the analysis of network evolution, this paper finds that the non-provincial cities like Xuchang and Foshan are in the second level with the help of Xuji Group Corporation and Midea Group, which shows the outstanding leading and driving ability of enterprises with strong innovation capability. In addition, the decline of Fushun in the evolution analysis indicates that resource-based cities have great difficulties in improving urban innovation ability.

## Comparative analysis of the proximity of urban innovation cooperation network

### Global spatial autocorrelation analysis

The global Moran’s I statistic can be exploited to identify the similarity of cooperation strength centrality among cities, which is conducive to grasp the phased evolution of China’s urban innovation cooperation as a whole. Based on the above analysis results, the GeoDA software was employed to calculate the GMI and its significance level of urban cooperation intensity in the three stages under the spatial proximity listed in Table 3. The principles of constructing spatial weight matrix are as follows. (1) Geographic weight matrix ($W_{ij}^{g}$). Due to the characteristics of urban distribution, this paper uses the KNN algorithm to determine whether the city is adjacent, and the default value of K is set to 4. (2) Network weight matrix ($W_{ij}^{n}$). Taking the average value of the urban network as the threshold, if the number of cooperation is greater than the average, $W_{ij}^{n}$ is equal to 1; otherwise, it is equal to 0. The mean value of the network matrix in 1985–2006, 2007–2015 and 2016–2017 is 0.2, 2.3 and 0.893, respectively.

**Table 3:** Global Moran’s I statistics and significance test value based on different proximity.

weight	statistics	1985–2006	2007–2015	2016–2017	1985–2017
Geographical weight	Moran’s I	0.033	0.048	0.044	0.047
	Z	1.3318	2.238	2.2536	2.4296
	P	0.063	0.06	0.045	0.045
network weight	Moran’s I	-0.413	-0.519	-0.407	-0.555
	Z	-16.3936	-26.4013	-30.9681	-39.8727
	P	0.001	0.001	0.001	0.001
pure network weight	Moran’s I	-0.491	-0.614	-0.466	-0.643
	Z	-20.547	-28.445	-36.3493	-45.359
	P	0.001	0.001	0.001	0.001

It can be seen from Table 3 that under the influence of geographical proximity, the innovation cooperation of Chinese cities presents a global spatial positive correlation with a significance level of 10% (GMI > 0). However, the first two stages fail to pass the 5% confidence level test, which indicates that the spatial difference of innovation cooperation of Chinese cities has certain randomness under the influence of geographical proximity. Besides, under the influence of network proximity, urban cooperation has a negative global spatial correlation (GMI<0), and the significance level of each stage was less than 1%. It shows that there is a close cooperative relationship between cities with different strength centrality, which confirms the conclusion that the urban network has a spatial structure of "core-periphery". The network proximity further consolidates the network system of urban innovation cooperation in China and shapes the spatial pattern by strengthening the cooperation between peripheral cities and core cities.

Comparing the effect of geographic proximity and network proximity on the strength centrality of innovation cooperation in Chinese cities, it is found that the cooperative relationship established through the network is more significant than the geographical proximity relationship within adjacent regions. Since it is difficult to directly compare GMI based on two different weights, the interaction effect of geographic proximity and network proximity directly affects the deviation of the calculation results. Therefore, referring to the research of Maggioni et al. [38], this paper separates geographic proximity from network proximity and defines "pure network proximity". Specifically, the geographic weight matrix is subtracted from the network weight matrix to construct the $w_{ij}^{g-n}$ weight matrix. Based on this, if there is a cooperation between two cities but they are not geographically adjacent, $w_{ij}^{g-n}$ is equal to 1; otherwise, it is 0. By comparing the results of network weights and pure network weights, it is found that the global spatial autocorrelation level under the pure network weights is more significantly negative than the normal one, which indicates the spatial autocorrelation and spatial dependence of network proximity in urban innovation cooperation in China.

### Local spatial autocorrelation analysis

**Fig 8:**
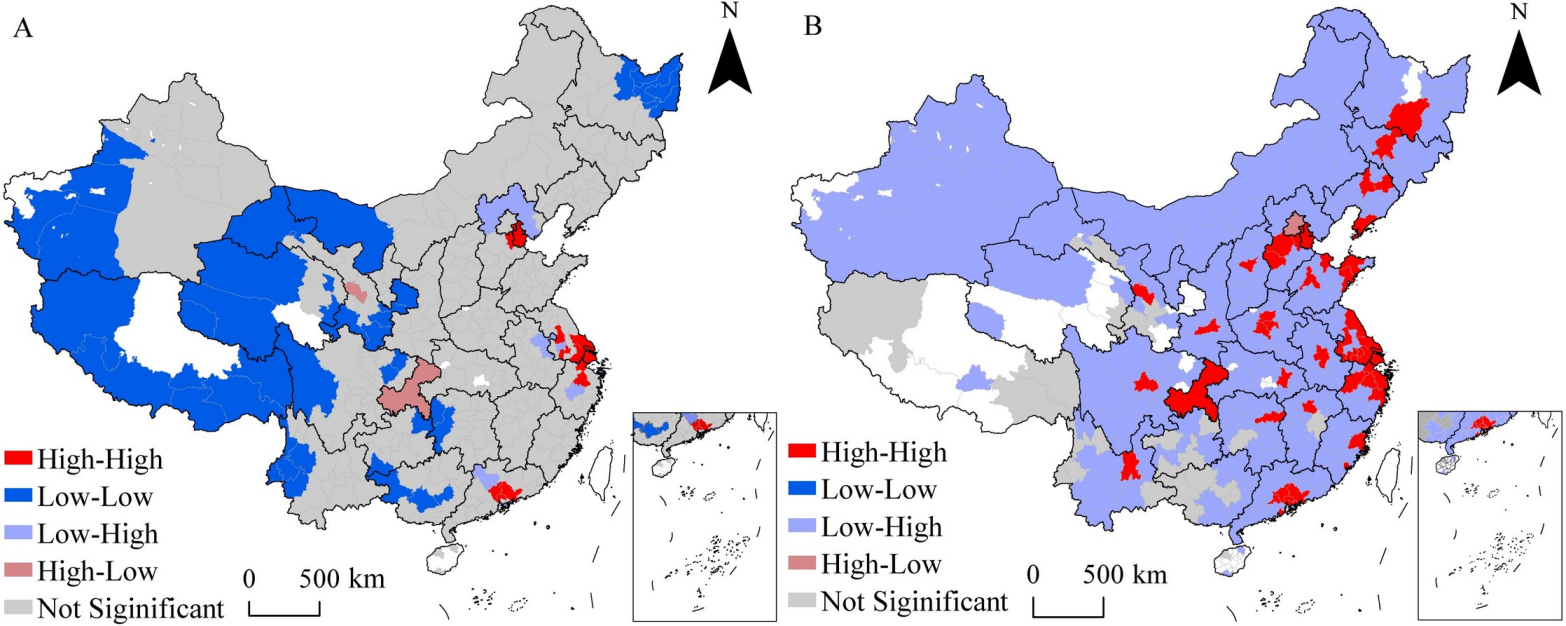
LISA cluster map of the strength degree of city co-innovation from 1985 to 2017. A. Geographic proximity. B. Network proximity. This map is drawn by the authors. This map was created using ArcGIS ® software by Esri, ©National Platform for Common Geospatial Information Services,Creative Commons Attribution 4.0(CC BY 4.0).

Compared with the global spatial autocorrelation analysis, the local autocorrelation analysis presents the spatial agglomeration or dispersion trend of innovation cooperation intensity between cities and their neighboring cities more clearly. (Fig 8) illustrates the LISA diagram of the strength centrality of innovation cooperation in Chinese cities at a significant level of 5% from 1985 to 2017.

It can be seen from (Fig 8A) that under the influence of geographical proximity, the significantly “High-High” agglomeration areas are mainly concentrated in the Beijing-Tianjin-Hebei Urban Agglomeration (Tianjin and Langfang), the Yangtze River Delta (Shanghai, Suzhou, Nantong, Jiaxing, etc.), and the Pearl River Delta (Guangzhou, Dongguan, Huizhou, etc.). The economic development of these regions is among the top in China. The urban agglomerations within these regions have a strong joint driving effect on attracting innovative resources. The “Low-Low” agglomeration areas are mainly concentrated in Xinjiang, Tibet, Qinghai, and other western regions of China, and Sichuan, Yunnan, and other southwestern regions, as well as Heilongjiang Province in northeast China. Lanzhou and Chongqing are both surrounded by cities with low cooperation strength centrality and belong to the significant “High-Low” agglomeration areas. These two cities are the growth poles of innovation cooperation in the western region, which is closely related to the high level of economic development, vast R&D investment, and extensive innovation resources of the cities themselves. Meanwhile, it is also related to the loss of talents and funds in the surrounding cities under the "siphon effect", which squeezes their ability to participate in innovation cooperation.

(Fig 8B) shows that under the influence of network proximity, the urban innovation cooperation has a significant network spillover effect, showing "High-High" and "Low-High" agglomeration characteristics. It should be noted that the analysis of network proximity should take the neighborhood structure between each city and other cities in the network cooperation relationship into consideration. The significantly “High-High” concentrated area presents the spatial pattern of "blossoming", which is mainly distributed in the provincial capital city, municipality directly under the central government, and vice provincial cities. This is mainly because the network proximity allows non-adjacent geographical regions to establish close cooperation, making local autocorrelation analysis focus more on the “High-High” cluster areas. The number of “Low-High” agglomeration areas accounts for 78% of the number of cities in the network, which is mainly because there are few national central cities in the network and most urban cooperation is limited to intra-regional cooperation. The absence of “Low-Low” agglomeration indicates that most marginal cities tend to establish links with core cities.

By comparing the Lisa diagram of the geographic proximity and network proximity, it can be found that the spatial agglomeration characteristics of urban innovation cooperation under network proximity ignore geographic proximity. According to the spatial and temporal pattern analysis on geographical proximity, the leading role of core cities cannot be well identified from the evaluation results. By contrast, the local spatial autocorrelation analysis on network proximity fully reflects the influence of "relational economy", and the evaluation results are clearer.

## Conclusions and discussion

### Conclusions

Based on the invention patents in China from 1985 to 2017, this paper analyzes the topological structure and spatial pattern of the overall and individual structure of China’s urban innovation cooperation network by applying the social network analysis method and taking cities at the prefecture level and above into consideration. Also, through the exploratory spatial analysis method, this paper compares and analyzes the different agglomeration characteristics of geographical proximity and network proximity. The analysis results are as follows:

(1) The development of China’s urban innovation cooperation network presents the characteristics of stages, and it has experienced the stages of initial development, rapid development, and gradual decline. This research result is not found in previous innovation research; The connectivity and centripetal concentration of urban innovation cooperation network are improved, and the imbalance needs to be further improved. The network has "quantity" development but lacks "quality" improvement; The degree of urban participation is gradually increasing, and the internal cooperation and external cooperation are improved simultaneously, consolidating the stability of the network structure.

(2) Consistent with the conclusion of other studies, the centrality of the urban innovation cooperation network also has obvious characteristics of administrative center orientation, coastal areas orientation, and ‘strong east and weak west’; Beijing is the center and bridge of the network. Beijing, Shanghai, Wuhan, Nanjing, and Guangzhou occupy the core hub position of the network in China. They undertake the functions of distribution and intermediary, and master the national innovation resources; A hierarchical "core-edge" structure is formed gradually for the urban innovation cooperation network, and the pyramid structure with Beijing standing at the top is being consolidated. The difference of conclusion lies in the comparative analysis of self-contained centrality and strength centrality that is concerned by this paper. The internal links of Shenzhen, Foshan, Qingdao, and other cities are stronger than the external links of these cities, and the external network links need to be improved. Besides, previous research has shown that Beijing, Shanghai, Guangzhou and Shenzhen are the core of the network[20]. However, this paper finds that after improving the quality of data screening, Beijing remains the core of the network, which indicates that the innovation level of Beijing is high.

(3) The geographic proximity shows a significant global spatial positive correlation, while the network proximity and pure network proximity have a more significant global spatial negative correlation. The network proximity further consolidates the network system of urban innovation cooperation in China by strengthening the cooperation between edge cities and core cities, and it shapes the spatial pattern of urban innovation cooperation. Based on network proximity, the local spatial autocorrelation of the Chinese urban innovation cooperation system is more obvious and recognizable, which better reflects the new development mode of "relational economy".

(4) Based on the above conclusions, we put forward some suggestions to optimize the urban innovation network in China.

Firstly, the network imbalance and Beijing’s "monopoly-dominate" network structure are not conducive to the sustainable development of China’s urban innovation network. It is necessary to strengthen the cultivation of secondary core cities, such as Shanghai, Wuhan, Nanjing, and Guangzhou, guide the coordinated development of large and medium-sized cities, and pay attention to improving the quality and efficiency of innovation ability.

Secondly, the location advantage, administrative advantage, and resource advantage are the key factors affecting the position of the urban innovation network. So, the coastal location advantage, administrative center advantage, and resource advantage should be fully utilized. Other cities can focus on cultivating and enhancing the polarization role of enterprises with strong innovation ability, establish a "national pipeline" between core enterprises and other cities, and then drive other innovation subjects in the city through "local buzzing" to give play to the core leading role of the city.

Finally, compared with geographical proximity, network proximity plays an increasingly important role. Strengthening the cooperation with cities close to the network may be an effective way for cities in remote areas to break regional lock-in and administrative barriers and improve their status in the urban innovation network.

### Discussion

Based on the study of the evolution of China’s urban innovation cooperation network, this paper explores the evolution characteristics of network structure and the role of different proximity, which provides a scientific basis for the formulation of China’s urban development strategy. However, there are still some limitations in this study. Firstly, the urban innovation cooperation network has the characteristics of complexity, diversity, and dynamics. Although the patent data exploited by this study is typical and representative, it is only a part of the representation of urban innovation cooperation capability. Therefore, it is necessary to involve the data of paper cooperation and scientific research project cooperation to improve the completeness of the data for the study. Secondly, geography is playing a more subtle role, which makes the choice of spatial scales essential. Although this paper and existing studies have achieved rich results at the national scale [10,14,17,19,20], the results obtained in a certain spatial scale may not be valid on another spatial scale [48]. In this case, the research on a single spatial scale cannot reflect the sensitivity of the structural characteristics of the innovation network to scale. Therefore, it is critical to reveal the different evolution characteristics of the innovation network from multiple spatial scales. However, due to limited space, it is impossible to discuss it in detail, which is also an important direction of future research.

## Supporting information

S1 Table Urban innovation cooperation network matrix. (XLSX)

## References

[1] Feldman MP, Audretsch DB. Innovation in Cities: Science-Based Diversity, Specialization and Localized Competition. Eur Econ Rev. 1999;43(2):409–29. doi: 10.1016/S0014-2921(98)00047-6

[2] Christaller W. The Central Places in Southern Germany. Upper Saddle River: Prentice Hall Press; 1966.

[3] Batten D. Network Cities: Creative Urban Agglomerations for the 21st Century. Urban Stud. 1995;32(2):313–27. doi: 10.1080/00420989550013103

[4] Meijers E. From central place to network model: theory and evidence of a paradigm change. Tijdschr Econ Soc Ge. 2007;98(2):245–59. doi: 10.1111/j.1467-9663.2007.00394.x

[5] Scott Allen J. Globalization and the Rise of City-regions. Eur Plan Stud. 2001;9(7):813–26. doi: 10.4324/9781315684871-31-3

[6] Derudder B, Taylor PJ. Central flow theory: comparative connectivities in the world-city network. Reg Stud. 2018;52(8):1029–40. doi: 10.1080/00343404.2017.1330538

[7] Taylor P. Specification of the World City Network. Geogr Anal. 2001;33(2):181–94. doi: 10.1111/j.1538-4632.2001.tb00443.x

[8] Li DD, Wang T, Wei YD, Yuan F. Spatial and temporal complexity of scientific knowledge network and technological knowledge network on China’s urban scale. Geographical Research. 2015;34(03):525–40. Chinese.

[9] Castells M. Grassrooting the space of flows. Urban Geogr. 1999;20(4):294–302. doi: 10.2747/0272-3638.20.4.294

[10] Wu K, Fang CL, Zhao MX. The spatial organization and structure complexity of Chinese intercity networks. Geographical Research. 2015;34(04):711–28. Chinese.

[11] Zhang WL, Chong ZH, Li XJ, Nie GB. Spatial patterns and determinant factors of population flow networks in China: Analysis on Tencent Location Big Data. Cities. 2020;99:102640. doi: 10.1016/j.cities.2020.102640

[12] Pan JH, Lai JB. Research on spatial pattern of population mobility among cities: A case study of "Tencent Migration" big data in "National Day–Mid-Autumn Festival" vacation. Geographical Research. 2019;38(07):1678–93. Chinese.

[13] You HL, Yang J, Xue B, Xiao XM, Xia JH, Jin C, et al. Spatial evolution of population change in Northeast China during 1992–2018. Sci Total Environ. 2021;776:146023. doi: 10.1016/j.scitotenv.2021.146023

[14] Zhang F, Ning Y, Lou X. The evolutionary mechanism of China’s urban network from 1997 to 2015: An analysis of air passenger flows. Cities. 2020;109:103005. doi: 10.1016/j.cities.2020.103005

[15] Wang JE, Mo HH, Wang FH, Jin FJ. Exploring the network structure and nodal centrality of China’s air transport network: A complex network approach. J Transp Geogr. 2011;19(4):712–21. doi: 10.1016/j.jtrangeo.2010.08.012

[16] Li CX, Gao X, He BJ, Wu JY, Wu KN. Coupling Coordination Relationships between Urban-industrial Land Use Efficiency and Accessibility of Highway Networks: Evidence from Beijing-Tianjin-Hebei Urban Agglomeration, China. Sustainability; 2019; 1446. Available from: https://www.mdpi.com/2071-1050/11/5/1446.

[17] Zhao XZ, Su J, Chao J, Liu XQ, Li TS, Rui Y, et al. The Character and Economic Preference of City Network of China: A Study Based on the Chinese Global Fortune 500 Enterprises. Complexity; 2020; 4312578. Available from: https://www.hindawi.com/journals/complexity/2020/4312578/.

[18] Alderson AS, Beckfield J, Sprague-Jones J. Intercity Relations and Globalisation: The Evolution of the Global Urban Hierarchy, 1981–2007. Urban Stud. 2010;47(9):1899–923. doi: 10.1177/0042098010372679

[19] Huang XD, Ma HT, Miao CH. Connectivity characteristics for city networks in China based on innovative enterprises. Acta Geogr Sin. 2021;76(04):835–52. Chinese.

[20] Duan DZ, Du DB, Chen Y, Zhai QH. Spatial-temporal Complexity and Growth Mechanism of City Innovation Network in China. Scientia Geographica Sinica. 2018;38(11):1759–68. Chinese.

[21] Han ZL, Yuan YY, Peng F. Research on the co- innovation network of government- industry- university of the equipment manufacturing industry in Northeast China. Economic Geography. 2018;38(01):103–11. Chinese.

[22] Ma HT, Li YC, Huang XD. Proximity and the evolving knowledge polycentricity of megalopolitan science: Evidence from China’s Guangdong-Hong Kong-Macao Greater Bay Area, 1990–2016. Urban Stud. 2020;Aug. doi: 10.1177/0042098020942665

[23] Wang QY, Zeng G, Lv GQ. Structural evolution of innovation networks of China’s equipment manufacturing industry. Acta Geogr Sin. 2016;71(02):251–64. Chinese.

[24] Ye Q, Xu X. Determining factors of cities’ centrality in the interregional innovation networks of China’s biomedical industry. Scientometrics. 2021;126(4):2801–19. doi: 10.1007/s11192-020-03853-3

[25] Gertler MS, Levitte YM. Local Nodes in Global Networks: The Geography of Knowledge Flows in Biotechnology Innovation. Ind Innov. 2005;12(4):487–507. doi: 10.1080/13662710500361981

[26] Torre A, Gilly J. On the Analytical Dimension of Proximity Dynamics. Reg Stud. 2000;34(2):169–80. doi: 10.1080/00343400050006087

[27] Krugman P R. Increasing Return and Economic Geography. J Polit Econ. 1991;99(3):483–99. doi: 10.2307/2937739

[28] Jaffe A. The Real Effects of Academic Research. Am Econ Rev. 1989;79(5):957–70.

[29] Asheim B, Coenen L, Vang J. Face-to-face, buzz, and knowledge bases: Sociospatial implications for learning, innovation, and innovation policy. Environment and Planning C: Government and Policy. 2007;25:655–70. doi: 10.1068/c0648

[30] Freeman C. Networks of innovators: A synthesis of research issues. Res Policy. 1991;20(5):499–514. doi: 10.1016/0048-7333(91)90072-X

[31] Cunningham SW, Werker C. Proximity and collaboration in European nanotechnology. Pap Reg Sci. 2012;91(4):723–42. doi: 10.1111/j.1435-5957.2012.00416.x

[32] Malmberg A, Maskell P. Localized Learning Revisited. Growth Change. 2006;37(1):1–18. 10.1111/j.1468-2257.2006.00302.x.

[33] Tijssen R, van de Klippe W, Yegros A. Localization, regionalization and globalization of university-business research co-operation in the United Kingdom. Pap Reg Sci; 2020; 1215–36. Available from: https://rsaiconnect.onlinelibrary.wiley.com/doi/10.1111/pirs.12531.

[34] Boschma R, Frenken K. The Spatial Evolution of Innovation Networks: A Proximity Perspective. In: Boschma R, Martin R, editors. The handbook of evolutionary economic geography. Cheltenham: Edward Elgar; 2010. pp. 120–35.

[35] Maskell P, Malmberg A. Localised learning and industrial competitiveness. Camb J Econ. 1999;23(2):167–85. doi: 10.1093/cje/23.2.167

[36] Marshall A. Principles of Economics. 1st ed. London: Palgrave Macmillan Press; 1890.

[37] Alpaydın UAR, Fitjar RD. Proximity across the distant worlds of university–industry collaborations. Pap Reg Sci; 2020; 689–711. Available from: https://rsaiconnect.onlinelibrary.wiley.com/doi/10.1111/pirs.12586.

[38] Maggioni MA, Nosvelli M, Uberti TE. Space Vs. Networks in the Geography of Innovation: A European Analysis. Pap Reg Sci. 2007;86(3):471–93. doi: 10.1111/j.1435-5957.2007.00130.x

[39] Bathelt H, Taylor M. Clusters, power and place: inequality and local growth in time–space. Geografiska Annaler: Series B, Human Geography. 2002;84(2):93–109. doi: 10.1111/j.0435-3684.2002.00116.x

[40] Fan CC, Scott AJ. Industrial Agglomeration and Development: A Survey of Spatial Economic Issues in East Asia and a Statistical Analysis of Chinese Regions. Econ Geogr. 2003;79(3):295–319. doi: 10.1111/j.1944-8287.2003.tb00213.x

[41] Frątczak-Müller J, Mielczarek-Żejmo A. Networks of cross-border cooperation in Europe–the interests and values. The case of Spree–Neisse–Bober Euroregion. Eur Plan Stud. 2020;28(1):8–34. doi: 10.1080/09654313.2019.1623972

[42] Asheim BT, Isaksen A. Regional Innovation Systems: The Integration of Local ‘Sticky’ and Global ‘Ubiquitous’ Knowledge. J Technol Transf. 2002;27(1):77–86. doi: 10.1023/A:1013100704794

[43] Balland P, Boschma R, Frenken K. Proximity and Innovation: From Statics to Dynamics. Reg Stud. 2015;49(6):907–20. doi: 10.1080/00343404.2014.883598

[44] Lee D. Towards Urban Resilience through Inter-City Networks of Co-Invention: A Case Study of U.S. Cities. Sustainability; 2018; 289. Available from: https://www.mdpi.com/2071-1050/10/2/289.

[45] Liu CL, Gui QC, Duan DZ, Yin MY. Structural heterogeneity and proximity mechanism of global scientific collaboration network based on co-authored papers. Acta Geogr Sin. 2017;72(04):737–52. Chinese.

[46] Freeman LC, Roeder D, Mulholland RR. Centrality in social networks: ii. experimental results. Soc Networks. 1979;2(2):119–41. doi: 10.1016/0378-8733(79)90002-9

[47] Rozenblat C. Opening the Black Box of Agglomeration Economies for Measuring Cities’Competitiveness through International Firm Networks. Urban Stud. 2010;47(13):2841–65. doi: 10.1177/0042098010377369

[48] Proost S, Thisse J. What Can Be Learned from Spatial Economics? J Econ Lit. 2019;57(3):575–643. doi: 10.1257/jel.20181414

